# Membrane of Plasma Rich in Growth Factors in Primary Pterygium Surgery Compared to Amniotic Membrane Transplantation and Conjunctival Autograft

**DOI:** 10.3390/jcm10235711

**Published:** 2021-12-06

**Authors:** Miriam Idoipe, Borja de la Sen-Corcuera, Ronald M. Sánchez-Ávila, Carmen Sánchez-Pérez, María Satué, Antonio Sánchez-Pérez, Gorka Orive, Francisco Muruzabal, Eduardo Anitua, Luis Pablo

**Affiliations:** 1 Ophthalmology Department, Miguel Servet University Hospital, 50009 Zaragoza, Spain; midoipe@gmail.com (M.I.); mamensp02@gmail.com (C.S.-P.); mariasatue@gmail.com (M.S.); asanch@telefonica.net (A.S.-P.); lpablo@unizar.es (L.P.); 2 Regenerative Medicine Laboratory, Biotechnology Institute (BTI), 01007 Vitoria, Spain; bdelasen@bti-health.com (B.d.l.S.-C.); gorka.orive@bti-implant.es (G.O.); francisco.muruzabal@bti-implant.es (F.M.); eduardoan-itua@eduardoanitua.com (E.A.); 3 Regenerative Medicine Laboratory, University Institute for Regenerative Medicine and Oral Implantology (UIRMI), 01007 Vitoria, Spain

**Keywords:** PRGF, pterygium surgery, amniotic membrane transplantation, conjunctival autograft, PRP, plasma rich in growth factors

## Abstract

This prospective and comparative study aimed to compare the use of a conjunctival autograft (CAG), plasma rich in growth factors fibrin membrane (mPRGF) or amniotic membrane transplantation (AMT) in primary pterygium surgery. Patients were assigned for surgery with CAG (group A), mPRGF (group B), or AMT (group C). Pterygium recurrence, Best Corrected Visual Acuity (BCVA), graft size (measured with anterior segment optical coherence tomography (AS-OCT)), and ocular surface symptoms (visual analogue scale (VAS) and ocular surface disease index (OSDI)) were evaluated. Thirteen eyes in group A, 26 in group B, and 10 in group C were evaluated. No changes in BCVA (*p* > 0.05) were found. Recurrence cases for groups A, B, and C were none, two, and two, respectively, and three cases of pyogenic granulomas in group A. The horizontal/vertical graft size was lower in group B vs group A (*p* < 0.05) from months 1 to 12. The improvement in VAS frequency for groups A, B, and C was: 35.5%, 86.2%, and 39.1%, respectively. The OSDI scale reduction for groups A, B, and C was: 12.7%, 39.0%, and 84.1%. The use of the three surgical techniques as a graft for primary pterygium surgery was safe and effective, showing similar results. The mPRGF graft represents an autologous novel approach for pterygium surgery.

## 1. Introduction

Pterygium is defined as a fibrovascular formation of triangular morphology that extends from the conjunctiva to the cornea [1]. This neoformation is more frequent in the nasal sector [1], and it is characterized by inflammation and fibrosis, leading to tissue remodeling [2]. Histopathologically, it affects the conjunctival collagen, leading to elastotic degeneration; Bowman´s membrane and corneal surface destruction are observed along with stem cell alterations [2]. A higher prevalence has been found in regions located thirty-seven degrees above and below the equator, with a higher ultraviolet (UV) intensity [1]. Di Girolamo et al. [3] showed that UV radiation stimulated the expression of matrix metalloproteinase (MMP)-1 in human ocular epithelial cells. Moreover, Nolan et al. [4] found overexpression of heparin-binding epidermal growth factor (HB-EGF) in pterygial tissue caused by UV radiation, which is considered as a driving force in the development of pterygium as it is a potent mitogen. Tsai et al. [5] highlighted in their study the importance of UV-mediated oxidative DNA damage in the formation of pterygium.

The prevalence of pterygium in the worldwide population is 12%, while in Spain, it is 5.9% (95% CI: 4.3–7.9) [6,7]. The most frequent symptoms are nonspecific due to tear film alteration (irritation, burning, photophobia, tearing, and foreign body sensation). Other less frequent and more specific symptoms are pain due to ulceration or decreased visual acuity because of corneal invasion [8]. Excision surgery is the only effective procedure in the treatment of pterygium. The usual procedures, according to complexity, are simple excision [8], excision with conjunctival autograft (CAG) [8,9,10], excision with an amniotic membrane (AM) graft [11], excision with mitomycin C [9,12,13,14], excision with limbal autograft [8], and lamellar sclerokeratoplasty [8]. Pterygium surgery with CAG remains the gold standard procedure, and involves placing the donor tissue using either suture or with a biological adhesive [15]. Autograft suturing requires surgical experience and technical skills. Suzuki et al. [16] reported that the use of silk or nylon sutures causes conjunctival inflammation and the migration of Langerhans cells in the cornea. Other drawbacks are increased surgical time, patient discomfort, Dellen, symblepharon, or graft rupture [17,18].

AM grafting is a widely used technique in ocular surface surgery [19]. Preserved human AM can be used as a substrate to replace damaged mucosal surfaces and successfully reconstruct the cornea [20] or conjunctival tissues after ocular surface neoplasia excision [21], as well as to repair scleral and corneal melting and perforations [22,23] and has been successfully included in pterygium surgery [24]. AM transplantation (AMT) improves ocular surface epithelialization, reducing inflammation, vascularization, and scarring [2,19]. However, some complications, including granulomas, superior anterior scars, symblepharon, and recurrences, have been found after using these surgical techniques [25,26]. Recently, numerous investigations have shown the relevance of platelets in regeneration processes by releasing biological mediators such as growth factors [27,28]. Plasma Rich in Growth Factors (PRGF) is a standardized type of platelet-rich plasma (PRP) with specific characteristics that differentiate it from other blood-derived products [29,30]. PRGF has been used in different medical fields [31,32,33]. Several properties of PRGF, in addition to its autologous origin and the absence of preservatives and stabilizers offers broad applicability in the ophthalmic field by using several formulations (eye drops, injectables, fibrin membranes or fibrin clots) [34,35,36].

Several studies have evaluated the features of PRGF in its eye drop formulation (ePRGF); it has been well-tolerated, demonstrating proliferative, cell migration, anti-inflammatory, antibacterial and antifibrotic capabilities [34,36,37,38]. Several studies have been carried out to evaluate the stability and safety of ePRGF during its storage. These studies showed that ePRGF maintains its biological activity after 12 months of storage under frozen conditions, for 7 days of daily, and use even stored at room temperature. No contamination was observed in any of the different storage conditions and temperatures analyzed [39]. On the other hand, the tolerance and usefulness of autologous PRGF fibrin membrane (mPRGF) in ophthalmology have been evaluated as an adjuvant to nonpenetrating deep sclerectomy [40] or ocular surface disorders [41] with positive results. This study aims to provide information about the safety and efficacy of mPRGF as a graft for pterygium surgery, compared with CAG and AM grafts.

## 2. Materials and Methods

This prospective, comparative, and observational clinical study evaluates the clinical results in pterygium surgeries carried out between February 2017 and April 2019 at Miguel Servet University Hospital, Zaragoza (Spain). This study was carried out following the principles of the Declaration of Helsinki, the regional clinical research ethics committee (Aragon, Spain) approved the conduct of this study (Authorization C.P.-C.I. EC16/0031). Informed consent forms were signed by all patients included in this study.

### 2.1. Patients

Patients included in this study had to be over 18 years of age and have a primary pterygium diagnosis requiring excision surgery (grade 2 or greater) [1]. Patients must have visual acuity greater than 0.5 Snellen (decimal). The exclusion criteria were: eyelid or conjunctival abnormalities (trichiasis, entropion, symblepharon), blepharitis, dry eye disease, persistent epithelial defects, glaucoma, and retinal or autoimmune diseases. Patients were consecutively assigned to one of these three groups: group A (surgery performed with CAG), group B (surgery performed with fibrin membrane obtained by PRGF technique), group C (surgery performed with an AM graft, with the basement membrane facing up). In the particular case of the patients in group B, coagulation issues and thrombocytopenia were included as exclusion criteria. The type of treatment received was known only by the surgeon performing the excision and was only revealed after the follow-up time had concluded. The clinical follow-up times were the same for the three groups: days 1, 7, 15, and months 1, 3, 6, and 12; this was carried out by a surgeon who did not know the group to which the patient belongs (single blind). Data from previous studies comparing the use of conjunctival autografts and amniotic membranes were analyzed [42,43], and the calculated sample size required to detect significant differences, assuming an alpha error of 5% and a beta error of 10%, was 50 patients, including 15 patients in group A, 25 patients in group B, and 10 patients in group C, with missing cases estimated to be 10%.

### 2.2. Outcome Measures

The primary outcomes were pterygium recurrence, Best Corrected Visual Acuity (BCVA), and graft size. The BCVA measured with Snellen optotype (decimal) was transformed to LogMAR (Logarithm of the Minimum Angle of Resolution), and the intraocular pressure (IOP) (mmHg) was measured with a Goldmann applanation tonometer. The pterygium grade was evaluated with a slit lamp [1]: grade 1 (atrophic), grade 2 (intermediate), and grade 3 (fleshy). The pterygium recurrence was evaluated using the Solomon scale [19]. Anterior segment optical coherence tomography (AS-OCT) (DRI OCT Triton^®^, Topcon Europe Medical B.V, Capelle aan den Ijssel, The Netherlands) was used for the baseline measurements of the pterygium size (µm): 1. thickness of the limbus, 2. horizontal size, 3. total horizontal size, and 4. vertical size. During the postoperative follow-up, the conjunctival restoration zone was also measured with AS-OCT (µm): 1. graft central thickness, 2. graft thickness in the limbus, 3. graft horizontal size (measured between the sclerocorneal limbus to the nasal area of the excised conjunctiva), and 4. graft vertical size. Symptoms related to alterations in the ocular surface were also evaluated, including the Visual Analogue Scale (VAS) for frequency and severity, and the OSDI scale (Ocular Surface Disease Index) [44]. The presence of a conjunctival defect or other complications were also evaluated.

### 2.3. PRGF Preparation

For the preparation of the autologous mPRGF and ePRGF, an Endoret^®^-PRGF^®^ ophthalmology kit (BTI Biotechnology Institute, S.L., Miñano, Alava, Spain) was used for each patient. Briefly, 50 mL of blood was extracted and processed following the protocol described by Anitua et al. [45]. Then, 12 mL of plasma was activated and incubated at 37 °C for one hour to obtain ePRGF. For the mPRGF preparation, fraction 2 (F2) (defined as 2 mL above the leukocyte layer) was used to obtain the mPRGF, while F1 (defined as the remaining plasma above F2) was discarded. For each membrane, 5 mL of F2 was activated and incubated at 37 °C for 20 minutes, and the obtained fibrin clot was conformed in a membrane shaper, obtaining mPRGF about 500 microns thick [41].

### 2.4. Surgical Procedures

**Figure 1:**
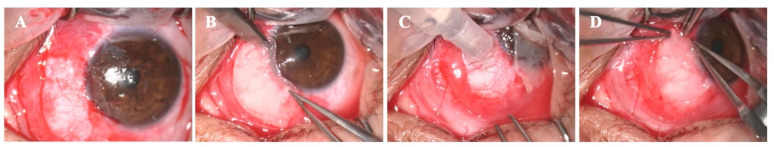
Use of mPRGF in pterygium surgery. (**A**) Bare sclera after pterigium surgery, (**B**) mPRGF was placed over the bare sclera and it was cut according to the size of the resected tissue, (**C**) one or two drops of fibrin glue was added to the bare sclera, and (**D**) mPRGF was placed over the fibrin glue, approximating the edges between the conjunctiva and the mPRGF to allow the gluing between them.

The surgeries were carried out by the same surgeon (MIC). After pterygium excision, different grafts were applied to each patient. (a) Conjunctiva autograft: the superior conjunctiva was used as a donor site for the graft. The graft was placed with the epithelial side up, and the limbal edge was positioned toward the limbus. Finally, fibrin glue (Tissucol^®^, Baxter AG, Vienna, Austria) was applied to fix the graft. (b) PRGF membrane: the membrane was placed over the bare scleral bed. Tissucol^®^ was applied at the scleral-PRGF membrane interface and held until complete gluing (see Figure 1). The patient received additional treatment with instilled ePRGF four times a day during the first month after the surgery. (c) Amniotic membrane graft: the AM was obtained from nonpreserved, lyophilized and cryopreserved samples on a cellulose nitrate filter. The epithelial/basement membrane side was positioned on the up side. Tissucol^®^ was applied at the scleral-AM interface, and the AM was held until complete gluing was achieved. The postoperative treatment in all groups was the same: Chloramphenicol and Dexamethasone eye ointment for 24-hour with eye bandage and then Dexamethasone/Tobramycin eye drops with a decreasing dosage for twelve weeks.

### 2.5. Statistical Analysis

The Kolmogorov–Smirnov test was performed to analyze the normal distribution. The continuous data were presented as mean, range, and standard deviation. A paired t-Student test or the Wilcoxon test was used to analyze the results obtained for all variables in each treatment group along the follow-up period. The ANOVA test, the Friedman test, or Cochran’s Q (in case of proportions) was used for repeated measurements. For independent data, ANOVA test (normal distribution) and a subsequent Bonferroni post hoc analysis for multiple comparisons between groups, a Kruskal–Wallis test in the case of nonnormal distribution, and chi-square tests were applied. The statistical program SPSS version 20.0 (SPSS Inc., Chicago, IL, USA) was used for the statistical analyses. A level of *p* < 0.05 was considered significant for all statistical analysis.

## 3. Results

Forty-nine eyes (49 patients) with primary pterygium were included, all of them were classified as grade 2 [1] and were divided into three groups: group A (13 eyes, 26.5%), group B (26 eyes, 53.1%), and group C (10 eyes, 20.4%). The country where patients had lived the longest is presented in Table 1. Demographic data are presented in Table 2.

### 3.1. Visual Acuity and Intraocular Pressure

**Figure 2:**
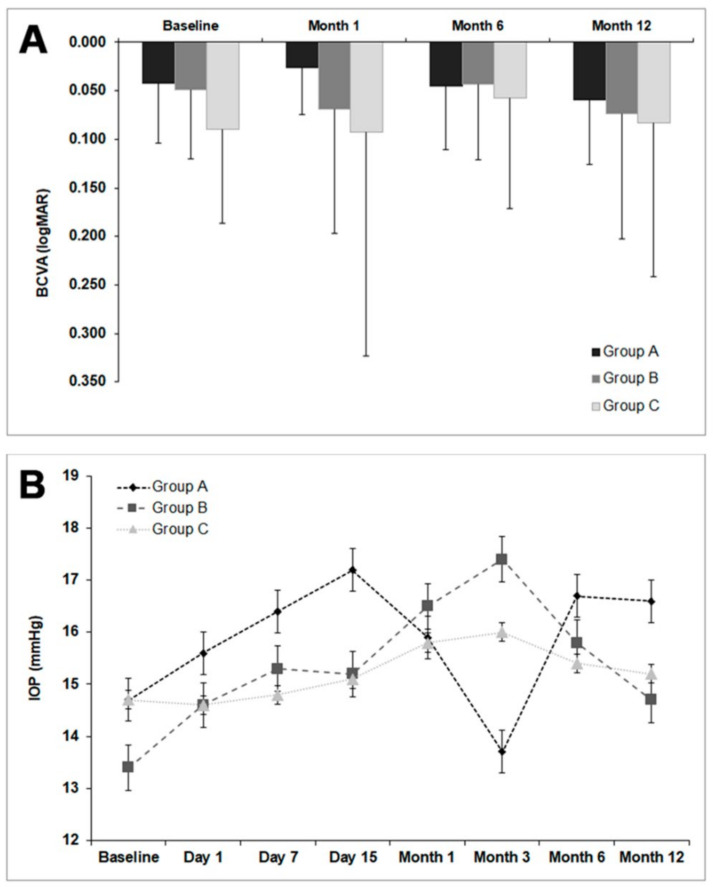
Visual acuity and intraocular pressure in the three treatment groups. (**A**) BCVA: best corrected visual acuity; Group A: conjunctival autograft; Group B: membrane - plasma rich in growth factors; Group C: amniotic membrane. No significant differences were found in visual acuity between the three groups (*p* > 0.05) during the follow-up time. (**B**). IOP: intraocular pressure; Group A: conjunctival autograft; Group B: membrane - plasma rich in growth factors; Group C: amniotic membrane. No significant differences were observed in IOP among the three groups (*p* > 0.05) during the entire follow-up time.

No changes in BCVA and IOP were observed between the baseline measurement and the end of each group’s follow-up (*p* > 0.05). Furthermore, there were no significant differences among the treatment groups for BVCA and IOP at any follow-up time (see Figure 2). The IOP was maintained between 13 and 18 mmHg during follow-up in the three groups.

### 3.2. Pterygium Measurement (AS-OCT)

On average, the mPRGF was reabsorbed at 13 days, while the AM was reabsorbed on average at 16 days. No statistical differences were obtained between the groups (*p* > 0.05) in the postsurgical baseline measurements for any of the variables analyzed (see Table 3). However, significant differences were found among the treatment groups in each variable analyzed with AS-OCT during the follow-up time (see Table 3).

**Figure 3:**
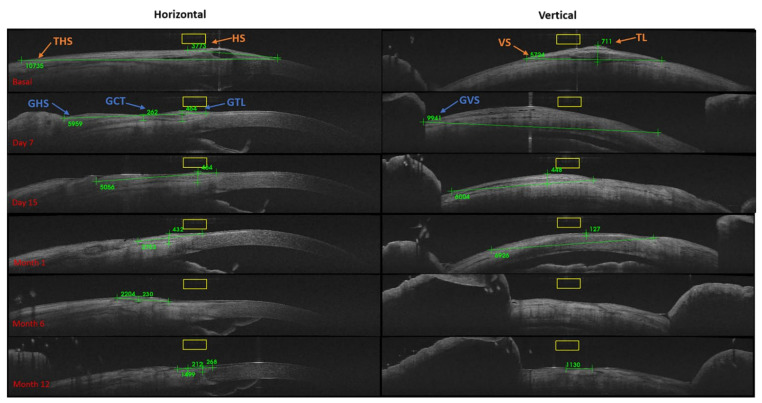
OCT graft measurements (µm) for mPRGF. Basal: TL: thickness of the limbus, HS: horizontal size, THS: total horizontal size, and VS: vertical size. During the postoperative follow-up, the conjunctival restoration zone was measured: GCT: graft central thickness, GTL: graft thickness in the limbus, GHS: graft horizontal size (measured between the sclerocorneal limbus to the nasal area of the excised conjunctiva), and GVS: graft vertical size.

Figure 3 shows representative images of a clinical case of a patient treated with a PRGF membrane graft, evaluating the follow-up by OCT, the mPRGF graft was reabsorbed by the second week. Subsequently, the evaluations of the graft size were related to the regeneration of the conjunctival epithelium (see Figure 3).

**Figure 4:**
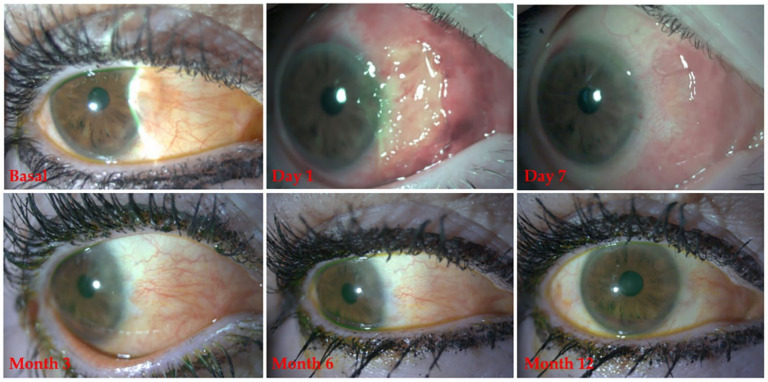
Clinical follow-up of a patient treated with mPRGF in pterygium surgery.

Figure 4 shows several images obtained from a patient treated with mPRGF as a graft showing a complete restoration of the conjunctiva during the follow-up period (see Figure 4).

### 3.3. Pterygium Recurrence: Solomon Scale

Group A showed lower pterygium recurrence throughout the follow-up, in contrast to groups B and C. Group A showed differences (*p* < 0.05) compared to group B at month 1 and 3, and showed differences compared to group C in month 6. No intraoperative complications were found in the three treatment groups. A descriptive analysis for recurrence (Solomon scale: grade 4), indicated two cases (7.7%) of the patients in the Group B (*n* = 26), and two cases (20.0%) of the patients in the Group C (*n* = 10). Moreover, during the first month of follow-up in group A, 3 cases with pyogenic granulomas were observed (see Table 4).

### 3.4. Ocular Surface Symptom Assessment (VAS and OSDI)

**Figure 5:**
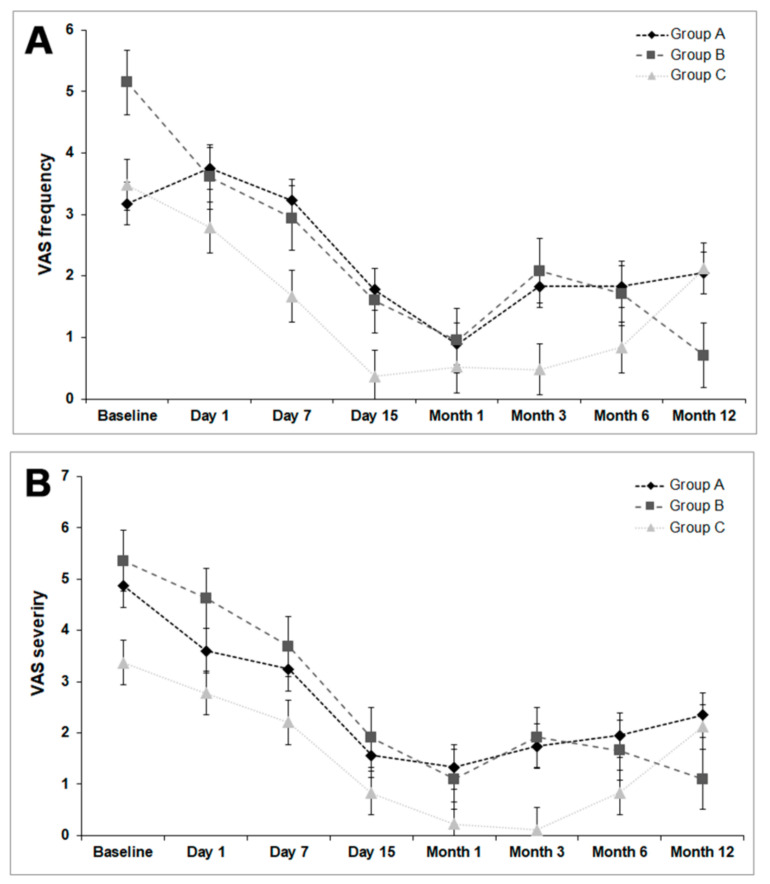
Visual analog scale. (**A**) Frequency. Group A: conjunctival autograft, Group B: membrane-plasma rich in growth factors, Group C: amniotic membrane. (**B**) Severity. Group A: conjunctival autograft, Group B: membrane-plasma rich in growth factors, Group C: amniotic membrane.

The VAS frequency and severity outcomes showed no significant differences (*p* >0.05) among the groups at any time of the follow-up time (see Figure 5). The improvement percentage in VAS frequency was 35.5% for group A, 86.2% for group B, and 39.1% for group C. The percentage improvement in VAS severity was 51.8% for group A, 79.5% for group B, and 37.1% for group C. The analysis of the OSDI questionnaire showed no significant differences among the different groups at any time of the study, except for group A and C, among which significant differences (*p* < 0.05) were observed at months 6 and 12 between both groups (see Table 5).

The reduction percentage in total OSDI score was 12.7% for group A, 39.0% for group B, and 84.1% for group C, and this change was significant (*p* < 0.05) in group B and group C, but not in group A (*p* > 0.05) (see Table 6). Group B showed significant differences (*p* < 0.05) in 6 symptoms (sensitivity to light, sensation of grit, eye pain, use of computer or screen, watching TV, and wind) with improvement in the OSDI score. However, group C showed only significant differences (*p* < 0.05) in two symptoms (blurred vision and low vision) and group A showed no differences (*p* > 0.05) in any symptoms.

## 4. Discussion

Surgical techniques for pterygium treatment have been improved over the years; nowadays, it is necessary to achieve the closure of the tissue defect, avoid recurrence, improve symptoms of the ocular surface, and increase life quality of patients [8,13,14]. To treat ocular defects and reduce the risk of ocular perforation, many techniques have been used in the past, including AMT, tissue adhesives (collagen, fibrin), animal-based tissue patches, limbal stem cell transplants, conjunctival autograft transplants or keratoplasty surgery [46,47]. The recurrence rate is the main result obtained in most clinical studies; meanwhile, the efficacy and safety results are evaluated using different surgical techniques. In our study, the primary outcome was pterygium recurrence, a fact that is consistent with the interests of current research [15].

In recent years, the field of ocular surface tissue regeneration has experienced significant progress. Some examples include the use of tissue replacements and auto-, allo- and xeno-grafts for limbal cell therapy, or pterygium surgery, either alone or in combination with a temporary graft such as an AM [48,49,50]. These grafts are not always useful, mainly due to the imbalance between demand and tissue availability and the immunological response between the donor tissue and the host [49,50]. Moreover, the use of allogeneic fibrin glues may potentially present certain biosafety risks, in the case of the AM, these risks will be enhanced due to its also allogeneic origin as one of its main disadvantages, along with the requirement of a tissue bank. Accordingly, using a safe and effective autologous tissue as a graft would be highly desirable, avoiding the risk of viral or prion transmission. In this sense, mPRGF provides a fibrin scaffold used as a regenerative and physical support membrane in many ocular defects. PRGF technology has a standardized protocol that guarantees the reproducibility of the treatment, the availability of direct costs related to its preparation and use, and immediate availability in the surgery room. Furthermore, it is also important to highlight that ePRGF is obtained during the same mPRGF preparation process and can be used as a postsurgical treatment, thus increasing the periodical availability of growth factors [34].

The main PRGF feature responsible for most of its biological effects is the sustained release of growth factors. However, the absence of leukocytes and antibacterial, anti-inflammatory, and anti-fibrotic activity are also essential characteristics of PRGF [35,51]. The growth factor release from the platelet´s alpha granules is mediated by calcium chloride, which activates fibrinogen and is converted to fibrin, and then begins to develop a three-dimensional acellular matrix with high stability [35,51]. Moreover, being mPRGF a leukocyte-free formulation potentially avoids faster fibrin degradation kinetics and a more significant proinflammatory response. It has been demonstrated that this mPRGF fibrin matrix retains trapped in the fibrin clot, almost 30% of the amount of growth factors remained trapped after eight days of incubation, for sustained release [35,51].

Nonetheless, this sustained release has shown an increment of the proliferation and migration activity of corneal keratocytes and conjunctival fibroblasts and the reduction of the TGF β1–induced myofibroblast differentiation reducing the number of α-SMA positive cells. This inhibition limits the fibrosis pathways, which is especially relevant in the pathogenesis of pterygium and its tissue remodeling [35,38,51,52]. Several studies have evaluated the potential benefits of mPRGF alone or in combination with other membranes like AM [34,41], showing a stable closure of the corneal defect in all patients treated with PRGF with no evidence of infection, inflammation, or pain [34,41].

In this study, no differences in BCVA and IOP were observed in the intergroup and intragroup analysis. In the anatomical evaluation, a progressive and sustained decrease in the size (horizontal and vertical) and thickness of the conjunctiva was observed in group B. In a study carried out by Zhang et al. [53] in 771 healthy subjects, a full conjunctiva thickness of 240.1 ± 29.8 μm was shown. In another study, the progression of the graft thickness in 40 pterygium surgery patients showed a graft thickness of 430 ± 127 μm in the primary pterygium group and 461 ± 178 μm in the recurrent group at one week after surgery and a graft thickness of 109 ± 15 μm and 107 ± 18 μm at month three, postoperatively [54]. The results obtained in the present study showed similar initial postsurgical thicknesses among the three groups for the graft placement area, most likely due to similar iatrogenic reasons in all procedures. All groups underwent a gradual size and thickness decrease, group B was the first to show graft thickness outcomes similar to those reported in healthy subjects at month 1 [53]. Moreover, there were significant differences between group B and the other two groups in month 3, with the mPRGF group achieving the lowest graft thickness outcomes. These findings might have been caused by a combined effect of autologous fibrin degradation and conjunctival tissue remodeling. When a fibrin graft is applied for wound healing purposes, it is invaded by surrounding cells, which will produce a new extracellular matrix to replace the fibrin meshwork, and the new tissue formation will be regulated by the gradual degradation of the fibrin clot (fibrinolytic process) [55]. The use of AS-OCT as a diagnostic and follow-up aid in ocular surface diseases such as pterygium or conjunctival tumors is increasingly common [56,57].

During the healing process in the mPRGF and AM groups, it is suggested that the degradation of the fibrin meshwork occurs, leading to a graft tissue replacement. A study carried out by Oscar Gris et al. [58] established that AM degradation may take a mean of 12.5 days (3 to 34 days). These results are similar to the use of mPRGF for the surgery of ocular surface disorders, in which complete mPRGF reabsorption occurred after a mean of 12.67 days [41]. Moreover, part of the fibroblast cells will be transformed into myofibroblasts during the wound healing process, favoring epithelial and endothelial cell migration through the graft and promoting wound contraction [59]. However, the persistence of myofibroblastic cells after wound healing could lead to the development of scarring tissue. Interestingly, it has been demonstrated that PRGF formulations reduce the number of myofibroblasts and modulate their action during wound healing, improving tissue regeneration and avoiding fibrosis formation [60,61,62]. Further studies are needed to determine the optimal graft size and degradation kinetics to avoid the risk of fast degradation that could compromise the pterygium surgery results.

The gold standard for pterygium surgery is excision with conjunctiva autograft, observing a recurrence between 1.9–8%. On the other hand, in a meta-analysis it was found that the graft with an amniotic membrane has greater recurrence (3.7–40.9%) than surgery with a conjunctival autograft (2.6–17.7%) [63]. In our study, for the Solomon scale, the overall results showed no statistical differences among the three groups at 12 months of follow-up (*p* > 0.05), showing that the three surgical techniques are similar in pterygium recurrence rates.

A clinical study with 108 patients comparing the use of platelet-rich fibrin (PRF) grafts and limbal conjunctival autografts (LCA) in pterygium surgery has recently been published, and it was observed that the surgery time was shorter in the PRF group (25.0 ± 4.2 min) than in the LCA group (36.5 ± 6.3 min) (*p* < 0.001) [64]. The use of mPRGF could decrease the surgical time compared to the conjunctival autograft group, since conjunctival dissection is not necessary; we believe that the surgical time would be similar to that of the amniotic membrane group.

In terms of ocular surface symptoms, the mPRGF group showed a higher percentage of improvement in VAS frequency (86.2%) and VAS severity (79.5%) compared to the other treatment groups. Similar results were observed in other studies treating several ocular surface diseases with PRGF, in which improvement of the VAS was demonstrated [34,36,37,38,41]. For the OSDI questionnaire, significant improvement was observed in the AM group than in the CAG group. However, no significant differences were showed between AM and mPRGF groups. Regarding the categories of symptoms, the mPRGF group obtained significant improvement (*p* < 0.05) in 6 of the twelve categories. However, the AM group only improved in 2 categories (*p* < 0.05), and the CAG group did not improve in any. One of the categories that improved with mPRGF treatment was eye pain. Several studies in different medical areas reported pain improvement after using PRGF [34,36]. The absence of leukocytes and endocannabinoid-mediated analgesic effects may be two of the main reasons for the pain reduction scores after PRGF treatment [35,65].

This study has some limitations, such as the fact that it was carried out at a single center, with a small cohort, and lacks inflammation biomarker measurements. Further studies are needed to determine the optimal surgical approach of mPRGF in graft placement and thickness. The results show that mPRGF is a safe and effective treatment for primary pterygium surgery, which produces an autologous graft in an agile way and contributes to preserve the patient´s healthy conjunctiva.

## 5. Conclusions

This is the first clinical study evaluating these three surgical techniques (CAG, AM, and mPRGF) to the best of our knowledge. The results obtained in this study suggest that the three evaluated techniques are effective in achieving tissue coverage. Therefore, mPRGF is a safe and effective treatment for primary pterygium surgery, allowing the production of an autologous graft quickly, without the need of a tissue bank and while avoiding iatrogenesis in healthy conjunctiva. This new surgical approach may be relevant for those cases of pterygium that require large excisions or with insufficient healthy conjunctiva.

## Figures and Tables

**Table 1:** The country where patients have lived the longest.

Country	Group A *n* (%)	Group B *n* (%)	Group C *n* (%)	Total *n* (%)
Ecuador	3 (6.1)	7 (14.3)	5 (10.2)	15 (30.6)
Spain *	1 (2.0)	7 (14.3)	2 (4.1)	10 (20.4)
Nicaragua	3 (6.1)	3 (6.1)	-	6 (12.2)
Colombia	2 (4.1)	2 (4.1)	-	4 (8.2)
Peru	1 (2.0)	1 (2.0)	1 (2.0)	3 (6.1)
Argelia	-	2 (4.1)	-	2 (4.1)
Brazil	1 (2.0)	-	-	1 (2.0)
Honduras	-	1 (2.0)	-	1 (2.0)
Romania	-	1 (2.0)	-	1 (2.0)
Senegal	1 (2.0)	-	-	1 (2.0)
Uruguay	-	1 (2.0)	-	1 (2.0)
Venezuela	-	-	1 (2.0)	1 (2.0)
Bolivia	-	-	1 (2.0)	1 (2.0)
France	1 (2.0)	-	-	1 (2.0)
Dominican Republic	-	1 (2.0)	-	1 (2.0)
Overall	13 (26.5)	26 (53.1)	10 (20.4)	49 (100)

* All from Zaragoza. Group A: conjunctival autograft. Group B: mPRGF. Group C: amniotic membrane.

**Table 2:** Demographics and sun protection.

	Group A	Group B	Group C	*p*-Value
Pacients, *n* (%)	13 (26.5)	26 (53.1)	10 (20.4)	-
Age, mean ± SD (range)	44.4 ± 12.9 (33.0–78.0)	47.5 ± 14.0 (31.0–77.0)	47.2 ± 14.0 (31.0–82.0)	0.791
Gender, M (%)	8 (61.5)	14 (53.8)	4 (40.0)	0.593
Race *				
Amerindian, *n* (%)	8 (61.5)	12 (46.2)	8 (80.0)	0.235
African, *n* (%)	1 (7.7)	3 (11.5)	0 (0.0)	
European, *n* (%)	4 (30.8)	11 (42.3)	2 (20.0)	
Evolution time of the pterygium, mean ± SD (range)	8.2 ± 4.6 (1.0–16.0)	6.1 ± 5.9 (1.0–30.0)	7.5 ± 5.5 (2.0–20.0)	0.503
Residence time in Zaragoza, mean ± SD (range)	16.2 ± 12.9 (1.0-–50.0)	20.2 ± 22.1 (0.0–75.0)	23.8 ± 23.4 (6.0–82.0)	0.668
Hours of sun exposure per day, mean ± SD (range)	2.52 ± 3.62 (0.0–10.0)	3.92 ± 3.39 (0.0–10.0)	2.29 ± 2.57 (0.0–8.0)	0.306
Sun protection *				
None, *n* (%)	5 (38.5)	14 (53.8)	3 (30.0)	0.429
Hat, *n* (%)	1 (7.7)	4 (15.4)	1 (10.0)	
UV filter glasses + Hat, *n* (%)	1 (7.7)	2 (7.7)	2 (20.0)	
UV filter glasses (occasional), *n* (%)	1 (7.7)	0 (0.0)	0 (0.0)	
UV filter glasses (usually), *n* (%)	5 (38.5)	4 (15.4)	4 (40.0)	

Group A: conjunctival autograft, Group B: mPRGF, Group C: amniotic membrane, M: male, SD: standard deviation, * Percentage calculated within each group, UV: ultraviolet.

**Table 3:** Graft sizes in each treatment group along the follow-up period measured by AS-OCT.

		Group A Mean ± SD (µm)	Group B Mean ± SD (µm)	Group C Mean ± SD (µm)	*p*-Value
Thickness next to limbus	Baseline	471 ± 143	478 ± 163	424 ± 110	0.767
Horizontal size from limbus		2862 ± 964	3469 ± 1594	2387 ± 988	0.106
Total horizontal size		7755 ± 2152	7482 ± 2817	7971 ± 1878	0.876
Vertical size in limbus		6206 ± 1236	6010 ± 1666	5343 ± 2038	0.461
Graft central thickness	Day 1	611 ± 216	455 ± 240	412 ± 207	0.103
	Day 7	620 ± 257 **	359 ± 271	452 ± 155	0.020
	Day 15	356 ± 141	316 ± 286	337 ± 119	0.362
	Month 1	324 ± 131	207 ± 163	271 ± 114	0.089
	Month 3	252 ± 147 **	84 ± 148	231 ± 147 ^#^	0.002
	Month 6	229 ± 90 **	102 ± 124	183 ± 45	0.011
	Month 12	151 ± 65	192 ± 109	190 ± 183	0.503
Graft thickness next to limbus	Day 1	459 ± 184	499 ± 417	412 ± 139	0.929
	Day 7	460 ± 232 *	248 ± 171	430 ± 183	0.005
	Day 15	365 ± 159	250 ± 193	274 ± 79	0.163
	Month 1	314 ± 114	259 ± 178	273 ± 109	0.770
	Month 3	250 ± 109 **	79 ± 118	230 ± 185 ^#^	0.002
	Month 6	207 ± 60 *	96 ± 115	188 ± 98	0.036
	Month 12	200 ± 91	197 ± 115	116 ± 37	0.226
Graft horizontal size	Day 1	6499 ± 2192	5949 ± 2336	5645 ± 2047	0.673
	Day 7	5813 ± 1894 *	3639 ± 2554	4784 ± 945	0.044
	Day 15	5287 ± 2411	3314 ± 2523	5983 ± 1028	0.059
	Month 1	4079 ± 1309 **	1048 ± 1829	3903 ± 903 ^#^	0.001
	Month 3	4598 ± 1492 **^‡^	1433 ± 2077	2626 ± 2346	0.003
	Month 6	3809 ± 1396 **	1433 ± 1908	2176 ± 1928	0.009
	Month 12	4815 ± 1426 *	1394 ± 1456	2299 ± 1970	0.003
Graft vertical size	Day 1	7356 ± 2322	7054 ± 2713	7882 ± 1153	0.651
	Day 7	7140 ± 2266	5230 ± 3640	6476 ± 1446	0.355
	Day 15	6298 ± 1619	5106 ± 3559	5983 ± 1097	0.442
	Month 1	6881 ± 959 **	4018 ± 3071	5320 ± 1906	0.007
	Month 3	6951 ± 1699 **^‡‡^	1649 ± 2450	3728 ± 2259	0.000
	Month 6	5926 ± 1274 **	1921 ± 2138	4762 ± 2507 ^#^	0.000
	Month 12	5653 ± 824 *^‡^	2374 ± 2457	2951 ± 1702	0.019

Group A: conjunctival autograft, Group B: mPRGF, Group C: amniotic membrane, SD: standard deviation. * Significant differences between group A and B, ** very significant differences between group A and B, ^‡^ significant differences between group A and C, ^‡‡^ very significant differences between group A and C. ^#^ Significant differences between group B and C.

**Table 4:** Solomon scale in the different treatment groups.

	Month 1 Mean ± SD	Month 3 Mean ± SD	Month 6 Mean ± SD	Month 12 Mean ± SD
Group A	1.00 ± 0.00 *	1.08 ± 0.29 *	1.10 ± 0.32 ^‡^	1.00 ± 0.00
Group B	1.55 ± 0.76	2.06 ± 0.87	1.88 ± 0.96	1.91 ± 1.04
Group C	1.11 ± 0.33	2.00 ± 1.22	2.43 ± 1.13	2.17 ± 0.98

Solomon scale: grade 1 (normal appearance of the surgery area), grade 2 (presence of some fine episcleral vessels without extending beyond the limbus, without any fibrous tissue in the excised area), grade 3 (presence of additional fibrous tissue in the excised area without invading the cornea, grade 4 (represents a true recurrence with fibrovascular tissue invading the cornea). Group A: conjunctival autograft, Group B: mPRGF, Group C: amniotic membrane. SD: standard deviation. * Significant differences between group A and B (*p* < 0.05), ^‡^ significant differences between group A and C (*p* < 0.05).

**Table 5:** Ocular surface disease index (OSDI) outcomes obtained in each treatment group at each time point of the follow-up.

	Baseline Mean ± SD	Day 7 Mean ± SD	Day 15 Mean ± SD	Month 1 Mean ± SD	Month 3 Mean ± SD	Month 6 Mean ± SD	Month 12 Mean ± SD
Group A	36.82 ± 26.00	36.45 ± 23.24	23.87 ± 18.61	19.60 ± 21.95	22.85 ± 22.85	31.31 ± 24.70	41.48 ± 27.34
Group B	33.92 ± 26.18	42.39 ± 25.60	26.62 ± 25.23	22.41 ± 21.76	20.04 ± 23.11	19.93 ± 23.46	20.68 ± 24.65
Group C	37.12 ± 26.18	38.85 ± 30.99	12.62 ± 14.79	5.72 ± 6.68	8.59 ± 14.51	7.87 ^‡^ ± 13.97	5.90 ^‡^ ± 9.72

Group A: Conjunctival autograft, Group B: mPRGF, Group C: amniotic membrane, SD: standard deviation. ^‡^ Significant differences between group A and C (*p* < 0.05).

**Table 6:** Results of the OSDI questionnaire: by symptom groups.

		Baseline Mean (Range)	Month 12 Mean (Range)	*p*-Value
Group A	Light sensitivity	2.08 (0–4)	2.00 (0–4)	0.41
	Grit feeling	2.15 (0–4)	1.50 (0–4)	0.34
	Eye pain	1.00 (0–4)	1.17 (0–4)	1.00
	Blurry vision	0.77 (0–3)	1.00 (0–2)	0.49
	Bad vision	0.69 (0–3)	1.00 (0–4)	0.59
	Read	1.08 (0–4)	2.50 (0–4)	0.24
	Night driving	0.88 (0–4)	0.40 (0–2)	0.32
	Use of computer or screen	0.92 (0–4)	1.33 (0–4)	0.85
	Watch TV	1.08 (0–4)	1.50 (0–4)	0.56
	Wind	2.38 (0–4)	2.83 (0–4)	0.79
	Very dry environments	2.46 (0–4)	2.50 (0–4)	1.00
	Air conditioning	1.92 (0–4)	1.83 (0–4)	0.79
	Total OSDI score	36.82 (0–93)	41.48 (6–61)	0.35
Group B	Light sensitivity	1.57 (0–4)	0.92 (0–3)	≤0.01 *
	Grit feeling	1.78 (0–4)	0.83 (0–3)	0.02 *
	Eye pain	0.91 (0–4)	0.67 (0–3)	≤0.01 *
	Blurry vision	0.83 (0–3)	1.80 (0–4)	0.19
	Bad vision	0.48 (0–3)	0.73 (0–3)	0.16
	Read	1.30 (0–4)	0.92 (0–4)	0.06
	Night driving	0.50 (0–4)	0.40 (0–3)	1.00
	Use of computer or screen	1.48 (0–4)	0.50 (0–3)	0.04 *
	Watch TV	1.22 (0–4)	0.83 (0–4)	0.03 *
	Wind	2.65 (0–4)	1.50 (0–4)	≤0.01 *
	Very dry environments	1.78 (0–4)	0.75 (0–3)	0.06
	Air conditioning	1.25 (0–4)	0.58 (0–3)	0.42
	Total OSDI score	33.9 (0–77)	20.7 (0–66)	≤0.01 *
Group C	Light sensitivity	1.50 (0–4)	0.33 (0–1)	0.11
	Grit feeling	2.30 (0–4)	0.67 (0–3)	0.06
	Eye pain	0.70 (0–3)	0.0 (0–0)	0.32
	Blurry vision	1.60 (0–4)	0.17 (0–1)	0.03 *
	Bad vision	1.60 (0–4)	0.17 (0–1)	0.03 *
	Read	1.00 (0–4)	0.17 (0–1)	1.00
	Night driving	1.00 (0–4)	0.00 (0–0)	1.00
	Use of computer or screen	0.78 (0–4)	0.20 (0–1)	0.32
	Watch TV	0.40 (0–4)	0.17 (0–1)	0.32
	Wind	2.40 (0–4)	0.50 (0–2)	0.07
	Very dry environments	2.40 (0–4)	0.17 (0–1)	0.10
	Air conditioning	1.50 (0–4)	0.17 (0–1)	0.10
	Total OSDI score	37.1 (6–75)	5.9 (0.25)	0.03 *

OSDI: ocular surface disease index; Group A: conjunctival autograft; Group B: mPRGF (membrane of plasma rich in growth factors); Group C: amniotic membrane; * *p* value < 0.05.

## Data Availability

The data used to support this study’s findings are available by contacting the corresponding author upon request.
